# The Abnormal CD4+T Lymphocyte Subset Distribution and Vbeta Repertoire in New-onset Rheumatoid Arthritis Can Be Modulated by Methotrexate Treament

**DOI:** 10.3390/cells8080871

**Published:** 2019-08-10

**Authors:** Jorge Monserrat, Cristina Bohórquez, Ana María Gómez Lahoz, Atusa Movasat, Ana Pérez, Lucía Ruíz, David Díaz, Luis Chara, Ana Isabel Sánchez, Fernando Albarrán, Ignacio Sanz, Melchor Álvarez-Mon

**Affiliations:** 1 Laboratory of Immune System Diseases, University of Alcalá, Alcalá de Henares, 28871 Madrid, Spain; 2 Department of Medicine, University Hospital “Príncipe de Asturias”, University of Alcalá and Instituto Ramón y Cajal de Investigación Sanitaria (IRYCIS), Alcalá de Henares, 28871 Madrid, Spain; 3 Immune System Diseases-Rheumatology Service, University Hospital “Príncipe de Asturias”, Alcalá de Henares, 28871 Madrid, Spain; 4 Division of Immunology and Rheumatology, Department of Medicine, Emory University, Atlanta, GA 30322, USA

**Keywords:** rheumatoid arthritis, CD4+ T lymphocytes, methotrexate, flow cytometry, TCR Vb

## Abstract

Patients with long-term, treated, rheumatoid arthritis (RA) show abnormalities in their circulating CD4+ T-lymphocytes, but whether this occurs in recently diagnosed naïve patients to disease-modifying drugs (DMARDs) is under discussion. These patients show heterogeneous clinical response to methotrexate (MTX) treatment. We have examined the count of circulating CD4+ T-lymphocytes, and their naïve (T_N_), central memory (T_CM_), effector memory (T_EM_) and effector (T_E_) subsets, CD28 expression and Vβ TCR repertoire distribution by polychromatic flow cytometry in a population of 68 DMARD-naïve recently diagnosed RA patients, before and after 3 and 6 months of MTX treatment. At pre-treatment baseline, patients showed an expansion of the counts of CD4+ T_N_, T_EM_, T_E_ and T_CM_ lymphocyte subsets, and of total CD4+CD28− cells and of the T_E_ subset with a different pattern of numbers in MTX responder and non-responders. The expansion of CD4+T_EM_ lymphocytes showed a predictive value of MTX non-response_._ MTX treatment was associated to different modifications in the counts of the CD4+ subsets and of the Vβ TCR repertoire family distribution and in the level of CD28 expression in responders and non-responders. In conclusion, the disturbance of CD4+ lymphocytes is already found in DMARD-naïve RA patients with different patterns of alterations in MTX responders and non-responders.

## 1. Introduction

Rheumatoid arthritis (RA) is a highly prevalent inflammatory arthritis. It afflicts 1% of the world’s population, reducing both the quality of life and life expectancy [1]. Fortunately, the last decades have seen great advances in the treatment of patients with RA. The possibility of controlling the progression of the disease, including the destruction of the affected joints, has improved through the use of methotrexate (MTX) and biological drugs with anti-tumor necrosis factor alpha (TNFα) activity [1,2]. Novel drugs and biological therapies are also becoming available, but MTX retains a central role in the treatment of RA and remains the most commonly used disease-modifying anti-rheumatic drug (DMARD) [3]. However, MTX fails to control disease activity and structural damage in some 30–40% of patients [4], and the precise mechanism of action of MTX in the treatment of RA remains unclear [5].

It is well established that the cells of the immune system play a pivotal role in the pathogenesis of the joint damage characteristic of RA, as well as in the extra-articular manifestations of the disease [1]. Although the mechanisms regulating this anomalous immune system response are not completely understood, considerable evidence supports that CD4^+^ T cells play a central role in initiating and perpetuating the chronic inflammation characteristic of RA [6,7,8].

CD4+ T lymphocytes are a phenotypically and functionally heterogeneous cell population. Based on their distinctive pattern of activation and effector functions, human CD4^+^ T cells can be divided according to the CCR7, CD27 or CD62L antigen surface expression into different subsets named differently in the literature. The main difference in the expression of these surface antigens is the kinetic of their loss along the different T activation/differentiation stages [9,10]. Therefore, it has been proposed that CD4^+^ T subsets include CD3^+^CD4^+^CD45RA^+^CCR7^+^ naïve (T_N_), CD3^+^CD4^+^CD45RA^−^CCR7^+^ central memory (T_CM_), CD3^+^CD4^+^CD45RA^−^CCR7^−^ effector memory (T_EM_) and CD3^+^CD4^+^CD45RA^+^CCR7^−^ effector (T_E_) subsets [11]. CD4+ T_N_ exhibit non effector function while CD4+ T_CM_ can rapidly proliferate and express multiple different effector molecules such as cytokines after being stimulated by antigen and diminished activation requirements [11,12,13]. CD4+ T_EM_ produce effector cytokines but have limited proliferative capacity and CD4+ T_E_ are at a final differentiation stage and share high levels of cytokine production [14]. The requirements for activation, proliferation and survival of these subsets are different, as well as their capacity to enter in lymphoid and inflamed non-lymphoid tissues [15].

Patients with RA show numerical and functional abnormalities in their CD4+ T lymphocytes both those in the circulation and those at inflamed joints [16,17,18,19,20]. Indeed, the anomalous differentiation of CD4+ naïve into CD4+ memory T lymphocytes, and imbalances between the different CD4+ T lymphocyte subsets, has been described with contradictory results [21,22,23,24,25]. Several factors might be involved in this variability, including the clinical stage of the disease and the previous and active treatments. Further, RA appears to be associated with the accelerated ageing of CD4+ T cells, with the expansion of those that no longer express the CD28 co-stimulatory molecule, and those which show telomere shortening [17,26]. The cause of this putative premature immune ageing remains unclear [27,28]. The results obtained in patients with long term disease might be secondary to the long-term maintained inflammatory stage of the disease and/or previous and active DMARD or immunosuppressor treatments.

The repertoire of the T cell receptor (TCR) plays a pivotal role in the antigenic self and non-self recognition and activation by CD4+ T lymphocytes [29]. Most of these cells have the alfa-beta type TCR and a given CD4+ T cell displays a single and unique combination of the variable (V), diversity (D) and joining (J) gene regions segments of the variable regions on its cytoplasmic membrane [30]. TCR repertoire represents the genetic background of the individual, in addition to the response to self or environmental antigens, being a very useful approach to understanding the antigen-expansion of T cells clones [31]. Variations in TCR repertoire have been found in several autoimmune diseases, and may be responsible for the breakdown of peripheral immune tolerance [32]. Heterogeneous results in the TCR repertoire have been described in RA patients [30,31,32,33,34,35,36,37,38,39,40,41,42,43].

To avoid the potential effects of long-term inflammation, DMARDs, other immunomodulatory drugs, and of concomitant disease on CD4+ T lymphocytes, the present work involved a homogenous group of patients recently diagnosed with RA, all of whom were DMARD-naïve. We used as controls a group of sex and age-matched healthy controls. The study of these patients might give information about the alterations of the CD4+ T lymphocytes in RA without the referred potential confusion factors. Furthermore, knowing which DMARD-naïve patients are likely and unlikely to benefit from MTX treatment before it begins would be advantageous for the selection of the treatment. The aim of the present work was therefore to examine the number and distribution of circulating T_N_, T_CM,_ T_EM_ and T_E_ CD4+ T lymphocyte subsets, their CD28 expression and their Vβ TCR repertoire distribution, before and after 3 and 6 months of MTX treatment in a homogenous group of recently diagnosed, DMARD-naïve patients with new-onset RA.

## 2. Materials and Methods

### 2.1. Inclusion and Exclusion Criteria

The study subjects were 68 Caucasian patients, all of whom fulfilled the American College of Rheumatology (ACR)/European League Against Rheumatism (EULAR) 2010 classification criteria for RA [44]. Patients were studied in parallel with 24 healthy sex-, age- and ethnicity-matched (proportionally) controls from areas of similar epidemiological background. The patients were monitored at the Immune System-Rheumatology Service, Hospital Universitario Príncipe de Asturias, Universidad de Alcalá, in Alcalá de Henares, Spain. All gave their informed consent to be included. The study was approved by the hospital’s clinical ethics committee.

### 2.2. Inclusion Criteria

Patients with new-onset RA (disease duration < 6 months), previously untreated with DMARDs were evaluated based on the following entry criteria: age ≥ 18 years, a diagnosis of RA and less than 3 months since the onset of RA clinical manifestations, a disease activity score of 28 (DAS28) according to EULAR criteria and DMARD-naïve status [44].

### 2.3. Exclusion Criteria

The exclusion criteria for this study included severe cardiovascular disease (congestive heart failure, uncontrolled hypertension, coronary disease, severe arrhythmia), hypercholesterolemia or diabetes mellitus; hematopoietic, lung, hepatic or renal disorders; active bacterial or viral infections; other autoimmune diseases; treatment with steroids, immunosuppressors or other drugs that interact with the immune system in the previous 6 months; possible pregnancy or lactation during the 6-month study period; simultaneous malignancy; malnutrition; and congenital immunodeficiency.

### 2.4. Study Protocol

All patients were treated weekly for 6 months with 15 mg oral MTX, adjusted by increments of 5 mg to 25–30 weekly until disease response criteria were met (or not in non-responders), plus 5 mg folic acid weekly. Patients were advised to take non-steroidal anti-inflammatory drugs at fixed doses during the study. All were monitored monthly to check for clinical and biochemical tolerance to MTX treatment, and at 3 and 6 months to assess the clinical response to treatment and to undertake immunological studies. Disease activity was determined using the DAS28 score according to EULAR criteria and the patient quality of life was measured using a validated Spanish version of the Health Assessment Questionnaire (HAQ) [45]. The clinical response of the patients to MTX treatment was defined according to EULAR criteria for RA [46], classifying patients as responders or non-responders. The responder group included those patients with a DAS28 score of <3.2, plus a DAS28 score after 6 months of MTX treatment that had fallen by at least 1.2 with respect to the initial score.

### 2.5. Isolation and Analysis of Peripheral Blood Mononuclear Cells

Three peripheral blood samples were taken from each patient by antecubital venipuncture at baseline (before starting MTX treatment) and at 3 months and 6 months after starting MTX treatment. Peripheral blood mononuclear cells (PBMC) were separated out by Ficoll-Hypaque (Lymphoprep^TM^, Axis-Shield, Oslo, Norway) gradient centrifugation [47]. They were then resuspended in RPMI 1640 medium (Biowhittaker Products, Verviers, Belgium) supplemented with 10% heat-inactivated fetal calf serum, 25 mM Hepes (Biowhittaker Products) and 1% penicillin-streptomycin (Biowhittaker Products). Cell enumeration was performed by conventional light microscopy using a Neubauer chamber, following trypan blue exclusion criteria for the identification of dead cells. The viability of fresh PBMC was checked by both trypan blue (light microscopy) and 7-aminoactinomycin D (7-AAD) (flow cytometry) exclusion.

**Figure 1:**
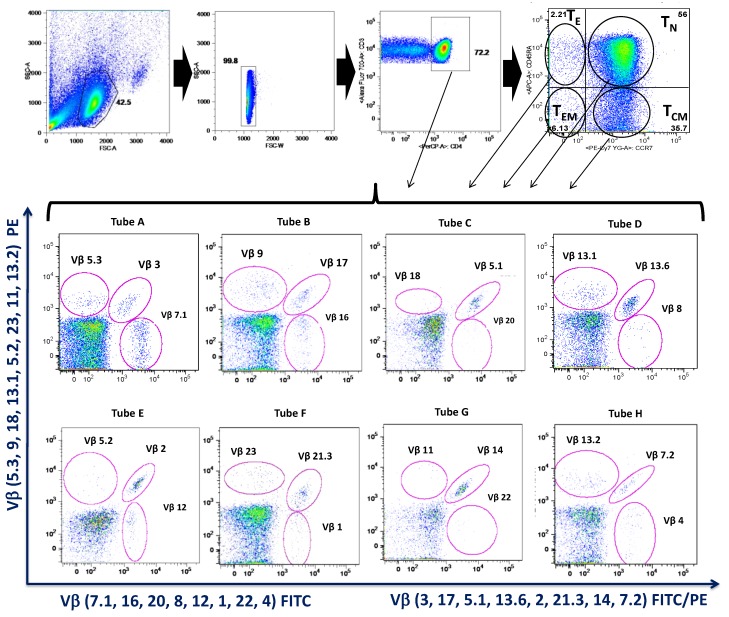
Study of the TCR Vβs repertoire gating strategy on CD4+ T lymphocytes. Note. Dot plots represent the gating strategy and all TCR Vβs repertoire evaluated in this study. Three Vβ are studied in FITC, PE or a combination of both fluorochromes in each activation/differentiation stages of CD4^+^ T lymphocytes.

T cells were phenotypically analyzed by nine-color polychromatic flow cytometry in a FACSAria-II flow cytometer running FACSDiva software (Becton-Dickinson, La Jolla, CA, USA). Two stained protocols were used. First, PBMCs were incubated with the next surface-labeled monocolonal antibodies CD3-Alexa700 (allophycocyanin Alexa 700, Becton-Dickinson), CD4-Percp (peridinin chlorophyll protein, Becton-Dickinson), CD45RA-APC (allophycocyanin, Becton-Dickinson), CCR7-PE-CY7 (phycoerythrin-cyanine 7, Becton-Dickinson) and CD28-FITC (fluorescein isothiocyanate, Becton-Dickinson) to study activation/differentiation stages of CD4+ T lymphocytes in combined of CD28 costimulatory receptor. Second, PBMCs were stained using labeled antibodies against the next surface antigens CD3, CD4, CD45RA and CCR7, CD3-Alexa700 (Becton-Dickinson), CD4-Percp (Becton-Dickinson), CD45RA-APC (Becton-Dickinson), CCR7-PE-CY7 (Becton-Dickinson) in combination with three Vβ (FITC, PE and FITC/PE, phycoerythrin, Beckman-Coulter International, Krefeld, Switzerland) until the 24 TCR Vβ analyzed were reached. Thus, the 24 Vβ studied were 7.1, 16, 20, 8, 12, 1, 22, 4 Vβ for FITC, 5.3, 9, 18, 13.1, 5.2, 23, 11, 13.2 Vβ for PE and 3, 17, 5.1, 13.6, 2, 21.3, 14, 7.2 Vβ for FITC/PE. A representative study of the gating strategy and the 24 TCR Vβ repertoire on CD4+ T lymphocytes and their activated stages by flow cytometry is shown in Figure 1.

All samples were acquired in a FacsAria-II flow cytometer with four lasers. The filter configuration and fluorochrome combinations were as follows: for the Violet laser (405 nm, 100 mW), Aqua probe (560/20); for the blue laser (488 nm, 100 mW) FITC (515/20) and PERCP (670/30); for the red yellow laser (561 nm, 100 mW) PE (582/15) and PE-CY7 (780/60); for the red laser (640 nm, 60 mW) APC (670/30); APC-Alexa 700 (730/45), APC-Alexa 750 (780/60). The staining protocol and quality and analysis controls were performed by ‘fluorescence minus one control’ as described by Roederer et al. [48]; the flow cytometry results are presented following the guidelines of the International Society of Advancement of Cytometry (ISAC) [49]. Samples were analyzed using FacsDiva 6.0 and Flow-Jo 7.0 software.

### 2.6. Statistical Analysis

Analyses were performed using SPSS-19 software (Statistical Package for the Social Sciences, SPSS-IBM, Armonk, NY, USA). Since most variables did not fulfill the normality hypothesis, the Mann-Whitney U-test for non-parametric data was used to analyze differences between groups, and analysis of variance followed by Wilcoxon tests was used for within group analyses. To assess the value of baseline circulating T CD4+ lymphocytes and their different subsets as predictors of MTX treatment response at baseline, 3 or 6 months after MTX treatment, receiver operating characteristic (ROC) curve analyses were performed, and the respective areas under the curves (AUC) determined. The best predictive cut-off value was defined as that which gave the highest product of sensitivity, specificity, positive predictive value (PPV) and negative predictive value (NPV). The significance level was set at *p* < 0.05.

## 3. Results

### 3.1. Patient Characteristics at Baseline

Table 1 shows the baseline characteristics of the recently diagnosed DMARD-naïve patients who eventually became responders (n = 48) or non-responders (n = 20) after six months of MTX treatment. No significant differences were seen in terms of age or sex distribution between these groups of patients with respect to any clinical or analytical variable examined. After six months of MTX treatment, the responders, however, showed a significant reduction in CRP (from 15.40 ± 6.51 mg/dL to 5.41 ± 2.52 mg/dL), in DAS28 (from 3.61 ± 0.62 to 2.35 ± 0.33), and in HAQ (from 0.82 ± 0.51 to 0.48 ± 0.42). The non-responders also showed a significant reduction in CRP (from 16.51 ± 6.11 mg/dL to 8.96 ± 4.21), but the reductions noted in DAS28 (from 3.75 ± 0.65 to 3.52 ± 0.29) and HAQ (from 0.81 ± 0.55 to 0.76 ± 0.66) were, however, not significant.

### 3.2. Recently Diagnosed, Dmard-Naïve Ar Patients Showed Increased Numbers of T_n_, T_em_ and Cd4+ T_e_ and Cd4+Cd28− T Lymphocytes,

At baseline, recently diagnosed DMARD-naive RA patients showed a significant increase in the circulating CD4+ T lymphocyte counts with respect to healthy controls (Table 2). This expansion of circulating CD4+ T lymphocyte is explained by a significant increase in the counts of CD4+ T_N_, T_EM_ and CD4+ T_E_ lymphocytes in recently diagnosed DMARD-naive RA patients. In addition, the number and percentage of circulating CD4+CD28− T lymphocytes was significantly increased in RA patients with respect to healthy controls. The main expansion of CD28− T cells was found in the CD4+ T_E_ subset from recently diagnosed DMARD-naive RA patients.

### 3.3. MTX Responder and Non-Responder Recently Diagnosed Ra Patients Showed Different CD4+ T Lymphocyte Distributions at Basal Conditions and after 3 and 6 Months of Treatment

**Figure 2:**
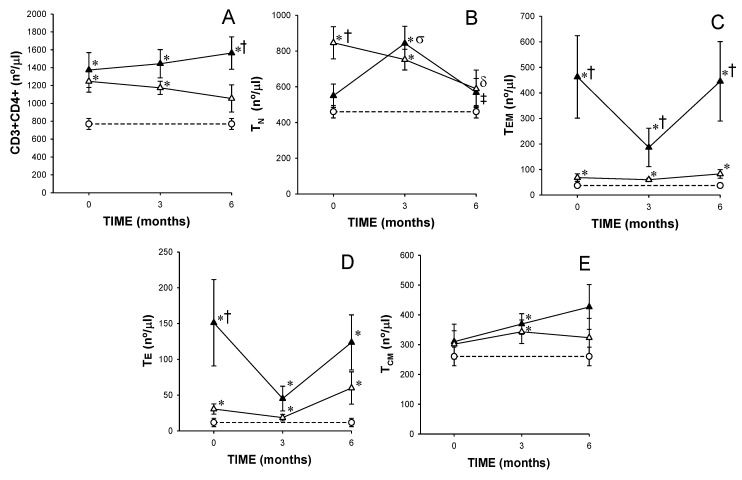
Panels A–E represents total and activation/differentiation stage subsets of CD4+ T lymphocytes in eventual responders and non-responders at baseline, and between responders and non-responders after 3 and 6 months of treatment. **Note**. Values represent numbers of naïve (T_N_), effector memory (T_EM_), effector (T_E_) and central memory (T_CM_) CD4^+^ T cells from (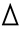) responders (including eventual at baseline) and (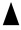) non-responders (including eventual at baseline) at up to 6 months of MTX treatment. The dotted line represents the mean of the results obtained for each variable in this group of subjects in the three different studies performed (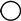). All values are expressed as mean cell counts ± S.E.M. *, *p* < 0.05 for responders or non-responders (including eventual) vs. healthy controls; †, *p* < 0.05 for responders vs. non-responders (including eventual), σ, *p* < 0.05 for values at 3 months of treatment compared to baseline, ‡, *p* < 0.05 at 6 months of treatment time compared to 3 months, and δ, *p* < 0.05 at 6 months of treatment time compared to baseline.

Before starting the MTX treatment, responder and non-responder patients showed a significant increase in circulating CD4+ T lymphocyte counts with respect to healthy donors but there were not significant differences between both groups of patients. However, after 6 months of MTX treatment, the non-responders showed a significant expansion of the CD4+ T lymphocyte population, and the responders normalized the CD4+ T lymphocyte counts (Figure 2A).

At baseline, responders showed a significant increase in the CD4+ T_N_ lymphocyte counts with respect to non-responders and healthy controls. After 6 months of MTX treatment, a significant reduction and normalization in the CD4+ T_N_ counts was found in responders. In the non-responders, a significantly larger number of CD4+ T_N_ cells were seen after 3 months of MTX treatment (Figure 2B).

At baseline and after 3 and 6 months of MTX treatment, the non-responders showed significantly larger CD4+ T_EM_ lymphocyte counts than responders and healthy donors. (Figure 2C). The CD4+ T_E_ lymphocyte counts were also significantly increased in both groups of patients with respect that found in healthy controls at baseline and after 3 and 6 months of MTX treatment. However, there were only significant differences in the CD4+ T_E_ lymphocyte counts between both groups of patients at baseline (Figure 2D). 

At baseline, responders and non-responders showed normal CD4+ T_CM_ lymphocyte counts. At 3 months, a significant increase in the CD4+ T_CM_ lymphocyte counts was observed in both groups of patients with respect to healthy controls (Figure 2E).

**Figure 3:**
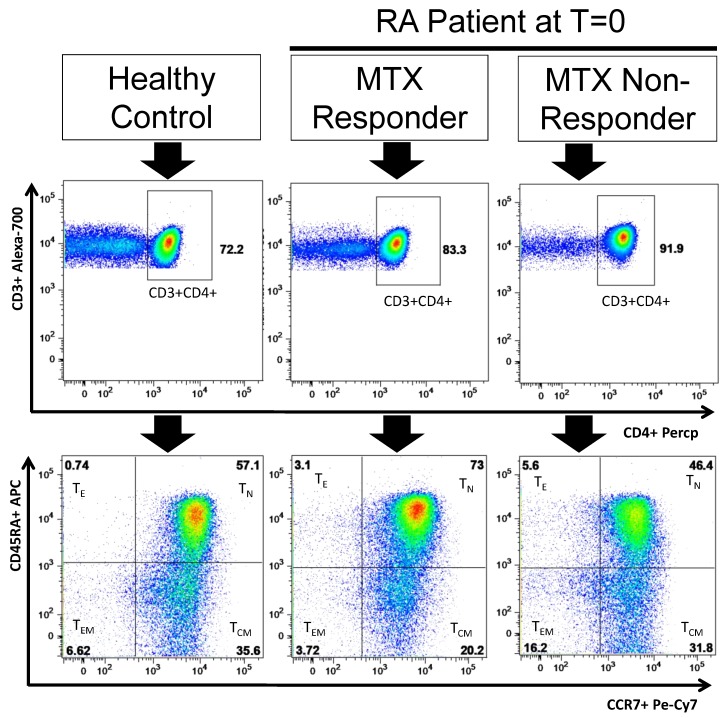
CD4^+^ T lymphocyte and their activation/differentiation stages in RA patients. **Note**. Dot plots represent the distribution of T CD4^+^ in percentages of naïve (T_N_), central memory (T_CM_), effector memory (T_EM_) and effector (T_E_) CD4^+^ T lymphocytes in a three representative situations: a healthy control, and a responder and non-responder RA patients at baseline of MTX treatment.

A representative dot plot of CD4+ T lymphocytes and their activation/differentiation stages expression from RA responder and non-responder patients at baseline and HCs is shown in Figure 3.

**Figure 4:**
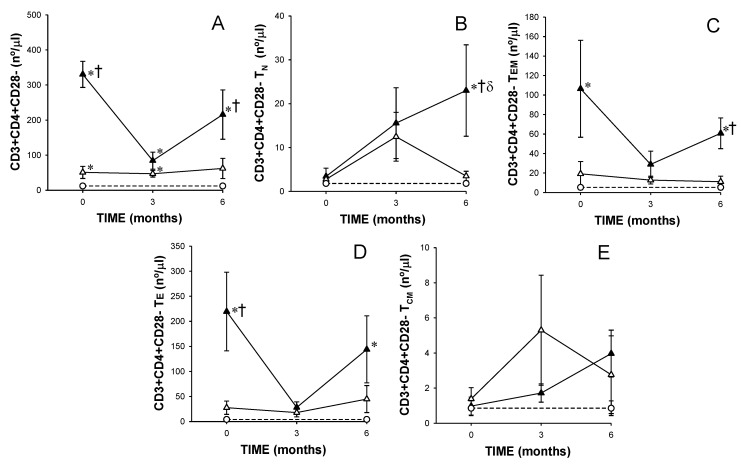
Panels A–E represents counts of CD4+CD28− T lymphocytes subsets in eventual responders and non-responders at baseline, and between responders and non-responders after 3 and 6 months of treatment. Note. Values represent numbers of total CD4+CD28− T lymphocytes and in their activation/differentiation stages (T_N_, T_EM_, T_E_ and T_CM_) from (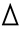) responders (including eventual at baseline) and (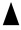) non-responders (including eventual at baseline) at up to 6 months of MTX treatment. The dotted line represents the mean of the results obtained for each variable in this group of subjects in the three different studies performed (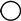). All values are expressed as mean cell counts ± S.E.M. *, *p* < 0.05 for responders or non-responders (including eventual) vs. healthy controls; †, *p* < 0.05 for responders vs. non-responders (including eventual), σ *p* < 0.05 for values at 3 months of treatment compared to baseline, ‡, *p* < 0.05 at 6 months of treatment time compared to 3 months, and δ, *p* < 0.05 at 6 months of treatment time compared to baseline.

The CD4+CD28− T lymphocyte counts were significantly increased in non-responders with respect to those found in responders and healthy controls at baseline (Figure 4A). However, non-responders showed a significant reduction in the CD4+CD28− T lymphocyte counts after 3 months of MTX treatment with a significant increase after 6 months of treatment. At baseline, non-responders showed a significant increase in the counts of CD4+CD28− T_EM_ and T_E_ with respect to responders (Figure 4C,D). However, after 6 months of treatment, non-responders had significant higher counts of CD4+CD28− T_N_, T_EM_ and T_E_ with respect to responders.

### 3.4. The Number of Circulating CD4+ T_EM_ Lymphocytes Predict the Clinical Response to MTX Treatment in Recently Diagnosed Dmard-Naive Patients

**Figure 5:**
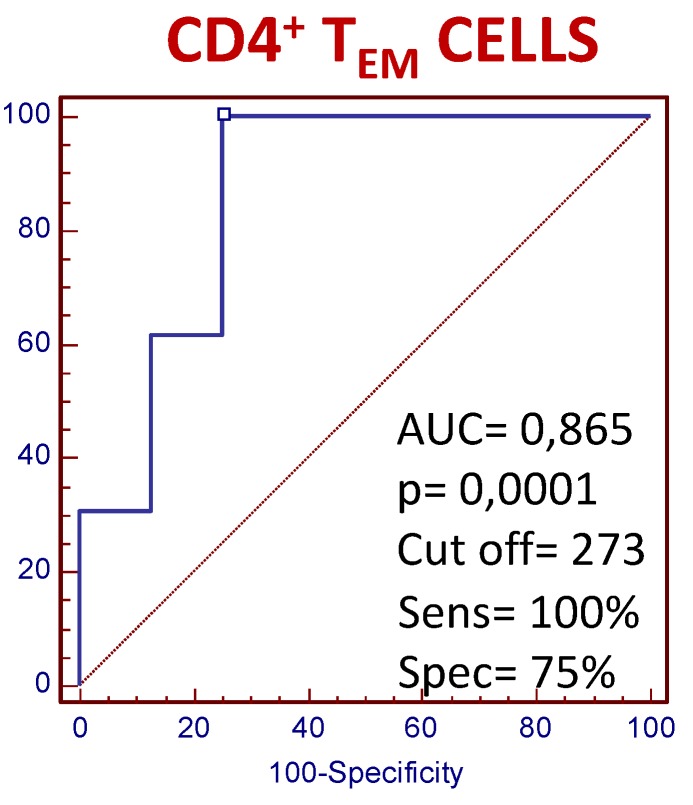
The receiver-operating characteristic (ROC) analysis of the counts of circulating CD4+ TEM lymphocytes at baseline of MTX treatment between responder and non-responder RA patients. Note. Receiver-operating characteristic (ROC) analysis of the counts of circulating CD4+ TEM lymphocytes. The predictive value of the absolute numbers of lymphocytes was determined by calculating the area under the curve (AUC). AUC and the optimum cut-offs (cells/µl) for distinguishing between MTX responders and non-responders, plus their sensitivity (Sens), specificity (Spec) values, are shown next to the curves. These were used to verify the validation of the ROC curves and to establish the predictive power of the cut-offs. The confidence intervals (C.I.) were 0.704 and 0.957.

Figure 5 shows the predictive value of the CD4+ T_EM_ lymphocyte counts with respect to clinical response to MTX. At baseline, a cut-off value of 273 cells/µl for circulating lymphocytes showed 100% sensitivity and 75% specificity in terms of discriminating between eventual responders and non-responders. The predictive values of the CD4+ T_N_, and T_E_ counts were inferior to those found for T_EM_ (data not shown).

### 3.5. MTX Responder and Non-Responder Recently Diagnosed Ar Patients Show Different Pattern of Distribution of the Vβ T-Cell Repertoire in Circulating CD4+ T Lymphocyte

**Figure 6:**
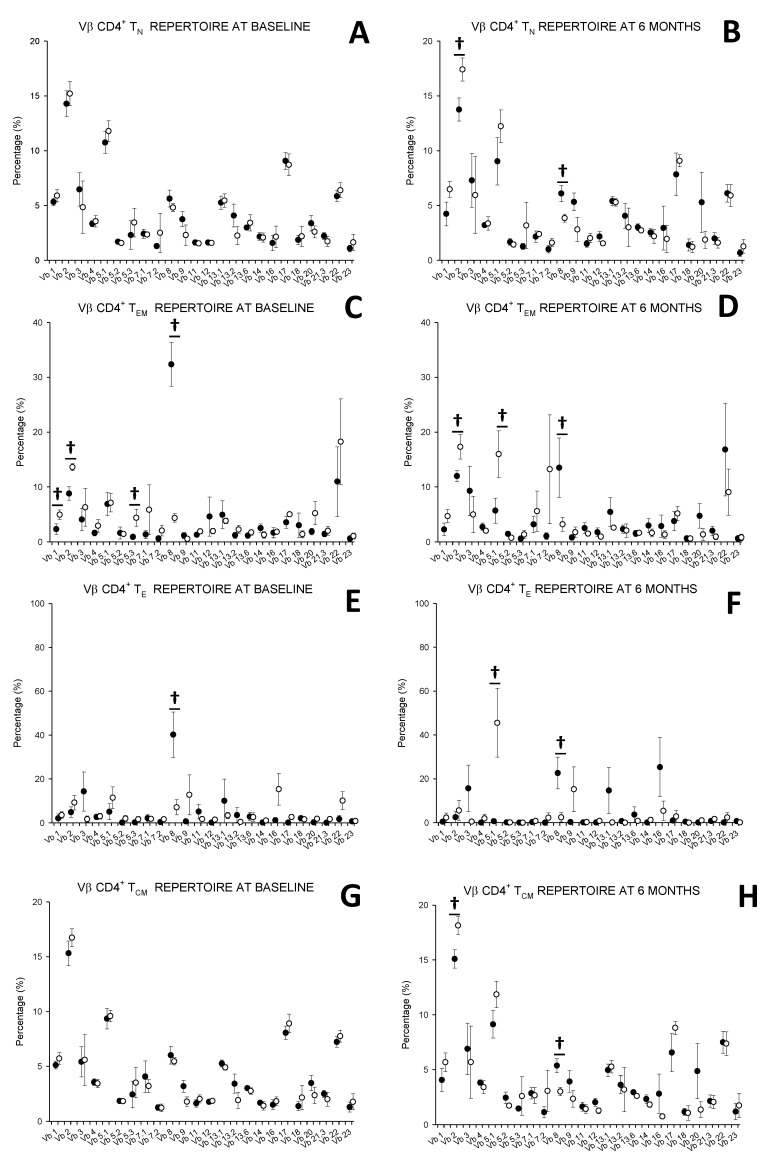
Panels A–H represents the TCR Vβ distribution of CD4^+^ T lymphocyte subsets in a responder and non-responder RA patients. **Note**. Data represent the TCR Vβ distribution of naïve (T_N_), effector memory (T_EM_), effector (T_E_) and central memory (T_CM_) CD4^+^ T cells from (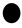)responders and (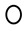) non-responders at up to 6 months of MTX treatment. All values are expressed as percentages ± S.E.M. †, *p*<0.05 for responders vs. non-responders.

We investigated the distribution of the 1, 2, 3, 4, 5.1, 5.2, 5.3, 7.1, 7.2, 8, 9, 11, 12, 13.1, 13.2, 13.6, 14, 16, 17, 18, 20, 21.3, 22 and 23 Vβ receptor T-cell repertoire families of the CD4+ T_N_, T_EM_, T_E_ and T_CM_ lymphocytes from MTX responder and non-responder recently diagnosed RA patients at baseline and after 6 months of treatment (Figure 6). We found an expansion of the Vβ8 TCR family in the CD4+ T_EM_ and T_E_ lymphocyte subsets at both times of the study in responders with respect to non-responders and in the T_N_ and T_CM_ after 6 months of treatment. However, non-responders showed increased percentages of Vβ2 TCR family in the T_EM_ subset at both times of the study and in T_N_ and T_CM_ subsets after 6 months of treatment. Furthermore, non-responders showed enhanced percentages of the Vβ1 and Vβ5.3 families in the CD4+ T_EM_ subsets at basal conditions and Vβ5.1 family in the CD4+ T_EM_ and T_E_ subsets.

**Figure 7:**
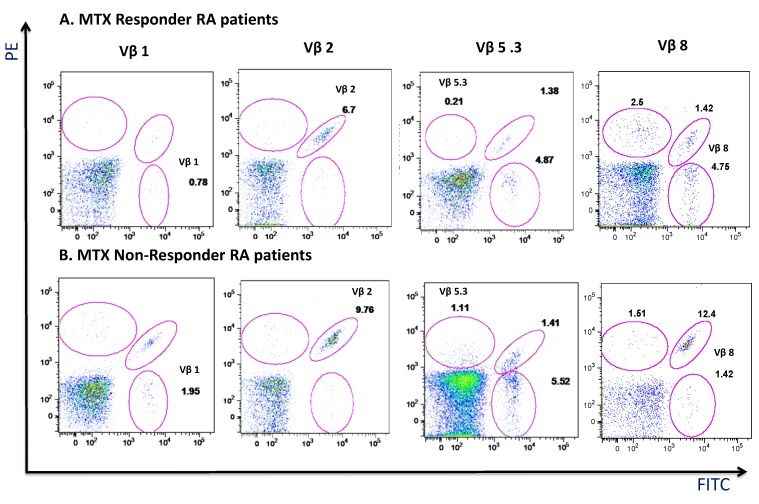
Representative dot-plot of Vβ expression in a responder and non-responder RA patients. Note. 1, 2, 5.3 and 8 Vβ repertoire distributions in CD4^+^ T_EM_ lymphocytes in a representative responder (**A**) and non-responder (**B**) RA patients at baseline of MTX treatment.

A representative dot-plot of significantly Vβ repertoire distributions in CD4^+^ T_EM_ lymphocytes in a responder (panel A) and non-responder (panel B) RA patients at baseline of MTX treatment is shown in Figure 7.

## 4. Discussion

The pathogenesis of RA remains unclear, but the immune system appears to play a pivotal role in the induction and maintenance of joint and extra-musculoskeletal manifestations of the disease [50]. Indeed, T lymphocytes seem to be involved in initial and continuing joint damage, as well as in extra-articular events [51]. We focused our translational study in the characterization of the CD4+ T lymphocytes in a homogenous group of recently diagnosed DMARD-naïve RA patients before starting MTX and along the first six months of treatment. Two main scientific reasons supported this research strategy. We selected a population of recently diagnosed and DMARD-naïve RA patients for allowing the identification of alterations in circulating CD4+ lymphocytes that may be ascribed to the pathophysiology of the disease avoiding the potential effects of long-term inflammation, DMARDs and other immunomodulatory drugs and/or comorbidities. Second, we hypothesized that the pattern of alterations in the CD4 lymphocytes in DMARD-naïve RA patients might condition the response to DMARD and have potential relevance as a predictive biomarker of therapeutic response. Our data show marked CD4+ T lymphocytes alterations in DMARD-naïve RA patients and two different populations can be identified by the pattern of activation/differentiation CD4+ T subset redistribution and CD28 expression. Interestingly, these two groups of RA patients show different clinical response to MTX treatment. The increased number of circulating CD4+ T lymphocytes MTX responders can be mainly ascribed to the expansion of the T_N_ but also show there is an increase in the counts of the minority T_EM_ and T_E_ subsets. The expansion of the T_N_ found in the MTX responders might be related to an increased production of these cells and/or to a reduction in their consumption explained by a diminished antigenic/inflammatory stimulation. In contrast, a dramatic expansion of T_EM_ and T_E_ subsets is found in MTX non-responders. Interestingly, CD4+ T_EM_ cells are characterized by their ability to enter inflamed non-lymphoid tissues [52,53,54]. Thus, the expansion of T_EM_ observed in DMARD-naïve RA patients and mainly in those MTX non-responders may contribute to their reported presence in the inflamed synovial of these patients [55]. The observation of a reduction in the counts of circulating T_N_ CD4+ lymphocytes in long-disease-duration and DMARD-treated patients has supported the knowledge of a deficient function of the thymus in RA patients [18,25]. However, our findings show a normal or increased number of T_N_ CD4+ lymphocytes in DMARD-naïve RA patients. These data do not support the involvement of the thymus deficiency in the initial stage of the pathogenesis of the RA. 

The different pattern of activation/differentiation CD4+ T subsets redistribution found in MTX responder and non-responder DMARD-naïve RA patients at basal conditions is differentially modified by the treatment. After six months of MTX treatment, non-responders remain with a marked expansion of the T_EM_ subset as well as of the T_EM_ cells conditioning the maintenance of increased counts of total circulating CD4+ lymphocytes. 

Previous studies of the activation/differentiation CD4+ T subset distribution in RA patients have shown conflicting results [21,22,23,24,25]. Several reasons may be involved in this heterogeneity such as differences in disease duration, previous and active DMARD use, immunosuppressor treatments, associated comorbidities, and genetic and epidemiological characteristics. As previously indicated, to reduce the impact of potential interferences with the mechanisms directly associated with RA pathophysiology, we investigated a clinically homogeneous population of recently diagnosed, DMARD-naïve patients with RA. Furthermore, the accuracy of the methodologies employed for the CD4+ T lymphocyte analysis have been improved such as those used in this study. Our results agree with the recently reported expansion of T_N_ and total T effector CD4+ in RA patients without DMARDs treatment in the previous three months to the study [56]. We have found that the pattern of activation/differentiation CD4+ T subset redistribution is different in MTX responders or non-responders at basal conditions and the variations observed along the treatment give a light to the understanding of the observed variation.

CD28 is a co-stimulatory molecule that plays multiple roles during T cell activation, proliferating, and survival [57]. A characteristic feature of long term activated and old T cells is the loss of the CD28 co-estimulatory receptor [57]. CD28 down regulation may result in reduced T-cell receptor activation and the release of certain proinflammatory cytokines [57,58]. Our results show clear differences in the numbers of CD28−CD4+ lymphocytes between both groups of responder and non-responder RA patients. At basal conditions, the DMARD-naïve patients of the MTX non-responder group show a selective marked expansion of the CD28-T_EM_ and T_E_ subsets and after 6 months of treatment are also observed in T_N_ and T_E_ subsets_._ In contrast, MTX responders show normal counts of CD28− T_N_, T_EM_, T_E_ and T_CM at_ basal conditions as naïve DMARD patients as well as after 6 months of MTX treatment. These results agree with previous observations of expanded CD28-CD4 T lymphocytes in early and long term treated RA [59,60]. However, our results do not support the knowledge of the expansion of the CD28-CD4 T lymphocytes as an initial finding of RA since it is only observed in a defined group of patients.

The differences in the CD4+ T lymphocyte compartment between MTX responder and non-responder RA patients is also supported by the study of their Vβ TCR repertoire. At basal conditions, MTX responders show an expansion of Vβ8 family expression on T_EM_ cells with respect non-responders and after 6 months also is expanded in T_N_ cells and T_MC_ subsets. In contrast, MTX non-responders show an expansion of Vβ1, 2, and 5.3 repertoire families in the T_EM_ subset with respect to responders in basal conditions. However, after six months of the MTX treatment, non-responders showed an expansion of Vβ2 in T_N_, T_EM_ and T_CM_, and Vβ5.1 in T_EM_ and T_E_. Thus, we have found different patterns of distribution of the Vβ TCR repertoire families between the activation/differentiation CD4+ T subsets in the MTX responder and non-responder patients and also along the evolution of the disease. These findings support the heterogeneity of the results described in previous studies of the Vβ TCR repertoire in CD4+ lymphocytes in RA [30,31,32,33,34,35,36,37,38,39,40,41,42,43]. Our findings indicate the analysis has to specifically study the different CD4+ subsets, the stage of disease evolution and the pattern of clinical response to the DMARD treatment for obtaining accurate results of the TCR repertoire in CD4+ lymphocytes form RA patients. Previous molecular biology studies performed in whole CD4+ lymphocytes preparations have not selectively analyzed the different CD4+ subsets [34,61,62,63,64]. It has been postulated that the Vβ8 family plays a pathogenic role in RA [36,41,65,66]. These studies have neither selectively analyze the expression of this family in the CD4+ subsets nor include DMARD naïve patients and compare the clinical evolution of the disease. Our findings of a clear expansion of Vβ8 family in T_EM_ and T_E_ subsets in basal conditions and also in T_N_ and T_CM_ after six months of treatment in MTX responder patients do not support the knowledge of the pathogenic role of this family in the first months of the disease. Moreover, an association between the expression of HLA-DR4 and the presence of the Vβ8 family has been found [67]. The expansion of the Vβ2 family has been reported in circulating and/or synovial fluid T lymphocytes or CD4+ population in a very limited number of RA patients [33,38,39].

Our data demonstrate that DMARD naïve RA patients have a disturbance of the CD4+ T lymphocytes but a different pattern of alteration is found in MTX responder and non-responders. The relevance of the differences in the CD4+ compartment found in both groups of patients is supported by the predictive value of MTX response of the CD4+ T_EM_ lymphocyte counts in DMARD naïve RA patients. These findings expand the knowledge of a different pattern of alteration of circulating immune cells in MTX responder and non-responder DMARDs naïve RA patients [68]. The cause of the different modification of the CD4+ lymphocytes found in both groups of patients along the first six months of treatment remains elusive. It might be related to a differential sensitivity of these cells to the action of MTX in both groups of patients and/or to the level of control of the inflammatory progression of the disease. The analysis of the spontaneous evolution of the CD4+ lymphocytes in untreated DMARD patients is not ethically supported. The long term follow up of the patients and the response to other treatments in MTX non-responders might give new clues for the understanding of the pathogenic and clinical relevance of the alterations observed in the CD4+ lymphocytes of RA patients at the first months of evolution and treatment. The study of a potential different pattern of CD4 alterations between MTX responders and non-responders to other DMARDs or biologic DMARDs is also of interest. It has been previously that monocyte populations are markers of response to adalimumab in MTX non-responder patients [69].

## Figures and Tables

**Table 1:** Patient demographic, clinical and biological characteristics at baseline.

Variables	Healthy Controls (n = 24) (mean ± SD)	Eventual Responders (n = 48) (mean ± SD)	Eventual Non-Responders (n = 20) (mean ± SD)
Age (years)	49.30 ± 8.40	51.05 ± 9.72	52.80 ± 9.75
Sex (women)	70.83%	72.91%	75.00%
CRP (mg/dL)		15.40 ± 6.51	16.51 ± 6.11
Rheumatoid factor (+)		90.76%	92185%
Anti-CCP (UI/mL)		425.61 ± 389.25	433.99 ± 295.60
DAS28		3.61 ± 0.62	3.75 ± 0.65
Erosions (+)		26.17%	27.07%
HAQ		0.82 ± 0.51	0.81 ± 0.55

CRP, C-reactive protein; anti-CCP, anti-cyclic citrullinated peptide antibody; DAS28, Disease Activity Score 28; HAQ, Health Assessment Questionnaire. The data represent the demography and clinical characteristics of RA patients according to their response to MTX at baseline of MTX treatment. All values are expressed as mean ± SD.

**Table 2:** Activation/differentiation stage of CD4 T lymphocyte subsets in the recently diagnosed DMARD-naïve patients at baseline.

n°/μL (%)	Controls	Patients at Baseline		Controls	Patients at Baseline
Subsets	CD4+			CD4+CD28−	
CD4+ Total (% with respect to total lymphocytes)	770.29 ± 61.59 (39.62 ± 3.07)	1265.96 ± 95.10 * (50.99 ± 2.24) *	(% with respect to total CD4+ lymphocytes)	12.16 ± 3.76 (1.72 ± 0.66)	101.77 ± 36.88 * (10.10 ± 4.14) *
T_N_(% with respect to total CD4+ lymphocytes)	460.55 ± 34.99 (60.61 ± 2.8)	737.13 ± 80.82 * (58.58 ± 4.00)	T_N_(% with respect to total CD4+CD28- lymphocytes)	1.8 ± 0.45 (0.40 ± 0.12)	2.87 ± 0.57 (0.7 ± 0.2)
T_EM_	37.44 ± 6.6 (4.63 ± 0.66)	164.43 ± 37.00 * (12.58 ± 3.07)	T_EM_	5.25 ± 2.31 (14.00 ± 4.5)	35.04 ± 19.03 (26.8 ± 5.6) *
T_E_	11.79 ± 5.85 (1.62 ± 0.71)	60.54 ± 17.46 * (5.40 ± 1.31) *	T_E_	4.24 ± 2.09 (46.89 ± 8.81)	62.54 ± 28.98 * (81.2 ± 4.50) *
T_CM_	260.5 ± 31.23 (33.14 ± 2.07)	303.84 ± 37.62 (23.42 ± 1.38) *	T_CM_	0.85 ± 0.41 (0.50 ± 0.30)	1.31 ± 0.52 (0.6 ± 0.30)

**Note.** Values represent numbers (cells/µL) and percentages (in brackets) of naïve (T_N_) effector memory (T_EM_), effector (T_E_) and central memory (T_CM_) activation/differentiation stages of CD4 + (Th) and CD4+CD28− T lymphocytes in the DMARD-naïve patients (n = 68) and healthy controls (n = 24) at baseline. % of CD4+ total are expressed with respect of total lymphocytes, % CD4+CD28− with respect of total CD4+ and T_N_, T_EM_, T_E_ y T_CM_ with respect of total CD4+ or CD4+CD28−, respectively. All values are expressed as mean ± S.E.M. * *p* < 0.05.

## Abbreviations

DAS28	Disease Activity Score 28
DMARDS	disease-modifying antirheumatic drugs
EULAR	European League Against Rheumatism
HAQ	Health Assessment Questionnaire
mAb	monoclonal antibodies
PBMC	peripheral blood mononuclear cells
FITC	fluorescein-isothiocyanate
PE	phycoerythrin
PerCP	peridinin chlorophyll protein conjugate
APC	allophycocyanin
APC-Alexa780	allophycocyanin-alexa-780
PE-CY7	phycoerythrin-cyanine seven
RA	rheumatoid arthritis
RPMI	Roswell Park Memorial Institute
SD	standard desviation
S.E.M.	standard error of the mean
